# Valproic acid sensitizes pancreatic cancer cells to natural killer cell-mediated lysis by upregulating MICA and MICB via the PI3K/Akt signaling pathway

**DOI:** 10.1186/1471-2407-14-370

**Published:** 2014-05-25

**Authors:** Pengfei Shi, Tao Yin, Feng Zhou, Pengfei Cui, Shanmiao Gou, Chunyou Wang

**Affiliations:** 1 Pancreatic Disease Institute, Department of General Surgery, Union Hospital, Tongji Medical College, Huazhong University of Science and Technology, 1277 Jiefang Avenue, Wuhan, Hubei Province 430022, P. R. China

**Keywords:** Valproic acid, Pancreatic cancer, Natural killer cells, MICA and MICB, PI3KCA protein

## Abstract

**Background:**

Valproic acid (VPA), a histone deacetylase (HDAC) inhibitor, is reported to exert anti-tumor effects by upregulating the expression of the natural killer group 2D (NKG2D) ligands on tumor cells; however, the mechanisms vary in different tumor types, and the effect and mechanism of action of VPA in pancreatic cancer cells are unknown.

**Methods:**

The present study evaluated the effect of VPA to susceptibility of pancreatic cancer cells to the NK cell-mediated lysis *in vitro* and *in vivo.* Then we investigated the mechanism which the effect of VPA depend on.

**Results:**

The lactate dehydrogenase assay (LDH) and xenograft experiment demonstrated that VPA significantly sensitized pancreatic cancer cells to NK cell-mediated lysis *in vitro* and *in vivo.* Quantitative real time- polymerase chain reaction (qRT-PCR) and flow cytometry demonstrated that VPA upregulated the mRNA and cell surface expression of the NKG2D ligands major histocompatibility complex class I-related chain A and B (MICA and MICB) in pancreatic cancer cells. Effects of VPA both *in vitro* and *in vivo* were significantly attenuated by the PI3K/Akt pathway inhibitor LY294002 or a siRNA targeting PI3K catalytic subunit alpha isoform (PI3KCA).

**Conclusion:**

VPA enhances the susceptibility of pancreatic cancer cells to NK cell-mediated cytotoxicity both *in vitro* and *in vivo* by upregulating the expression of MICA and MICB via a PI3K/Akt signaling pathway-dependent mechanism.

## Background

Pancreatic cancer remains a deadly and as yet incurable disease, with a five-year survival rate below 5% [1]. The poor prognosis of patients with pancreatic cancer is due to the high frequency of diagnosis at a late stage of disease and the lack of effective therapeutic methods [2]. Therefore, novel therapeutic strategies are urgently required for the treatment of pancreatic cancer.

Natural killer (NK) cells are a component of the innate immune response and contribute substantially to the anti-tumor immune response [3]. The anti-tumor immune response has gained significant attention in adoptive immunotherapy techniques for cancer [4]. The immune effects of NK cells are dependent on the natural killer group 2D (NKG2D)-mediated cell kill, and the efficiency of NKG2D-mediated cytotoxicity has been shown to correlate with the expression levels of NKG2D ligands (NKG2DLs) on the target cells [5]. However, tumor cells are able to escape from NKG2D-mediated immune surveillance by shedding MHC class I chain related (MIC) molecules from the tumor cell membrane [6,7]. Therefore, identification of a method to upregulate the expression of NKG2DLs on tumor cells would have a major impact on the efficacy of NK cell-mediated immunotherapy.

Valproic acid (VPA), a histone deacetylase inhibitor, is commonly used as an anti-epileptic drug. Recently, VPA was reported to induce apoptosis [8,9] in a variety of solid tumor types including glioma [10], neuroblastoma [11], breast cancer [12], colon cancer [13], and hepatocarcinoma [14], but not in non-malignant cells, which suggests that VPA may have potential as an anti-cancer treatment. Although VPA has been reported to induce a wide range of biological effects via various mechanisms, its ability to mediate the expression of NKG2DLs is considered to be an important component of its anti-tumor effect [15-17]. The interactions between NKG2D, expressed on the surface of immunocytes, and its ligands expressed on the surface of tumor cells are required for effective NK cell-mediated cytotoxicity. Increasing the expression of NKG2DLs on the surface of tumor cells has been documented to promote the anti-tumor effects of immunocytes. The MHC class I chain-related sequence A (MICA) and the MHC class I chain-related sequence B (MICB) are well-characterized NKG2DLs, and play an important role in NK cell-mediated anti-tumor immune responses [18]. It was previously reported that VPA enhances NK cell-mediated cytotoxicity in myeloma, ovarian, and liver cancer cells by increasing the expression of MICA and MICB; however, the mechanisms responsible for this effect vary depending on the tumor type [17,19,20].

So far, the effect and mechanisms action of VPA in pancreatic cancer remain unclear. In order to explore whether VPA has potential as a treatment for pancreatic cancer, we examined the effects and mechanism of VPA action on the expression of MICA and MICB in human pancreatic cancer cells. Our data demonstrates that VPA enhances the susceptibility of pancreatic cancer cells to NK cell-mediated cytotoxicity both *in vitro* and *in vivo* by upregulating the expression of MICA and MICB via activation of the PI3K/Akt pathway.

## Methods

### Patients and samples

Seventy-eight patients with pancreatic ductal adenocarcinoma (PDAC) underwent surgical treatment in Pancreatic Disease Institute, Union Hospital (Wuhan, China) during June 2012 and December 2012 (aged between 33 and 79; median age, 56 years; 45 males and 33 females). The surgical specimens were studied retrospectively. The samples were fixed in 4% formalin solution for 18-24 hours and embedded in paraffin for immunohistochemical analysis. The diagnosis of all patients was confirmed by histologic examination. The use of the clinical samples for analysis was approved by the Ethics Committee of Huazhong University of Science and Technology.

### Reagents and antibodies

Sodium valproate (VPA) and interleukin-2 was obtained from Sigma-Aldrich, St. Louis, MO, USA. Bovine serum albumin (BSA) and trypsin were purchased from Amresco, Solon, OH, USA. Fetal bovine serum (FBS), donor equine serum (DES), Alpha modified eagle medium (alpha-MEM), and Dulbecco’s modified eagle medium F12 (DMEM/F12) were obtained from Hyclone, Logan, UT, USA. Lapatinib, LY294002, rabbit polyclonal antibodies against PI3KCA, Akt Rabbit mAb, Phospho-Akt (Ser473) Rabbit mAb, HER3 Rabbit mAb, Phospho-HER3 Rabbit mAb, GAPDH Rabbit mAb, and goat anti-rabbit IgG antibodies conjugated to HRP were purchased from Cell Signaling Technology, Danvers, MA, USA. Anti-NKG2D mAb was obtained from R&D, Minneapolis, MN, USA. Phycoerythrin (PE)-labeled antibodies against human MICA and MICB and mouse IgG1 isotype control antibody were obtained from Biolegend, San Diego, CA, USA. Rabbit polyclonal antibodies against MICA and MICB were obtained from Santa Cruz, Santa Cruz, CA, USA.

### Cell culture

The human pancreatic adenocarcinoma cell lines PANC-1, MIA PaCa-2, and BxPC-3, and the human natural killer cell line NK-92 were obtained from the American Type Culture Collection (ATCC; Manassas, VA, USA). PANC-1, MIA PaCa-2 and BxPC-3 cells were cultured in DMEM/F12 containing 10% FBS. NK-92 cells were maintained in alpha-MEM containing 12.5% DES, 12.5% FBS, and 10 ng/mL interleukin-2. All cells were cultured in incubator at 37°C in a 5% CO_2_ atmosphere.

### Flow cytometry

PANC-1, MIA PaCa-2, and BxPC-3 cells were cultured to 80-90% confluence, trypsinized, washed twice with phosphate buffer solution (PBS), re-suspended in PBS at 1 × 10^6^ cells/100 μl, incubated with PE-anti-human MICA and MICB antibody or an isotype control antibody for 30 min, and then analyzed on a Becton Dickson LSR II flow cytometer (BD, Franklin Lakes, NJ, USA).

### Quantitative real-time RT-PCR

Total RNA was extracted from PANC-1, MIA PaCa-2, and BxPC-3 cells using TRIzol reagent (Invitrogen, Carlsbad, CA, USA) and reverse transcribed using SuperScript VILO cDNA Synthesis Kit (Invitrogen). The expression of human epidermal growth factor receptor 2 (HER2), human epidermal growth factor receptor 3 (HER3), ataxia telangiectasia mutated kinase (ATM), ATM- and Rad3-related kinase (ATR), MICA, MICB, PI3KCA, and β-actin were quantified using the quantitative SYBR Green PCR kit (TaKaRa Bio) according to the manufacturer’s protocol. The primers used for qRT-PCR are shown in Additional file 1: Table S1.

### Western blotting

Whole cell extracts were prepared using RIPA lysis buffer containing 1 mM PMSF, and the protein concentrations of the supernatants were determined using the BCA protein assay kit (Thermo Scientific, Rockford, IL, USA) according to the manufacturer’s protocol. Western blots were performed following standard procedures. Densitometry was performed using Image J V.1.46r (National Institute of Health).

### Small interfering RNA-mediated knockdown of PI3KCA

A siRNA targeting human *PI3KCA* (si-PI3KCA) was purchased from Ribobio, Guangzhou, China; a scrambled siRNA was used as a negative control (NC). PANC-1 and BxPC-3 cells were plated in 24-well plates and transfected using Lipofectamine 2000 (Invitrogen) according to the manufacturer’s instructions. The siRNA sequences are shown in Additional file 1: Table S2.

### Cellular cytotoxicity assay

Cytolytic activity was assayed using the standard lactate dehydrogenase (LDH) release assay. The target PANC-1, MIA PaCa-2, and BxPC-3 cells were incubated with or without 1 mM VPA for 24 h, washed, NK-92 cells were added to the target cells as effector cells, and the cells were co-cultured for 4 h at 37°C. To block NKG2D on NK-92 cells, 10 μg/ml anti-NKG2D mAb or mouse IgG1 isotype control antibody were added to the NK cells 30 min before co-culture. Spontaneous release of LDH by the target cells alone was < 15% of the maximal release of LDH by target cells lysed in 1% NP-40. The experimental LDH release values were corrected by subtraction of the spontaneous LDH release values of effector cells at the same dilution. Percentage lysis was calculated as: (corrected experimental LDH release - spontaneous LDH release) / (maximum LDH release – spontaneous LDH release) × 100.

### Xenograft experiment

Four-week-old female NOD/SCID mice were randomly divided into four groups (n = 5 per group) for each pancreatic cancer cell lines. The mice in the control group were subcutaneously injected into the flank with 2 × 10^6^ untreated PANC-1 cells or BxPC-3 cells, and the mice in the three experimental groups (NK, NK + VPA, and NK + VPA + LY294002) were co-injected with 2 × 10^6^ PANC-1 cells or BxPC-3 cells and 1 × 10^7^ NK-92 cells, and then repeatedly injected with 1 × 10^7^ NK-92 cells at the same site every 2 days during the experiment. The NK + VPA and NK + VPA + LY294002 groups were injected with PANC-1 cells or BxPC-3 cells which had been pre-incubated with 1 mM VPA for 24 hours and were intraperitoneally injected with 500 mg/kg VPA every 2 days during the experiment; the NK + VPA + LY294002 group were also intraperitoneally injected with 25 mg/kg LY294002 every 2 days during the experiment. Tumor volume was calculated every week using the formula: length × width^2^ × 0.5. The mice were sacrificed 4 weeks after the initial injection and the xenografts were excised and subjected to immunohistochemical analysis. All experimental protocols were approved by the Committee on the Ethics of Animal Experiments of the Union Hospital, Huazhong University of Science and Technology.

### Immunohistochemistry

Sections (4 μm) were prepared from the paraffin-embedded human primary tumors and mouse xenograft tumors. Immunohistochemistries were performed following standard procedures. For mouse xenograft tumors, the positive cells were counted, and the percentage was calculated. For clinical specimens, MICA and MICB expression were scored semi-quantitatively on the basis of the staining intensity and percentage of positive cells. Samples with less than 20% positive cells was considered to be weak expression, while that with more than 20% positive cells was considered to be strong expression.

### Statistical analysis

Data were presented as the mean ± standard deviation for flow cytometry, quantitative real-time RT-PCR, western blotting, cellular cytotoxicity assay, and xenograft assay, analyzed by t-test. Data of clinical characteristics were analyzed by Chi-square test. A significance threshold of P < 0.05 was used. Data were analyzed using SPSS v.11 statistical software (SPSS, Inc.).

## Results

### MICA and MICB expression was related to the clinical characteristics of pancreatic cancer

Immunohistochemistry analysis revealed the MICA and MICB expression in pancreatic cancer (Additional file 2: Figure S1). The expression of MICA and MICB in pancreatic cancer was significantly correlated with late TNM stage, tumor differentiation and lymphatic invasion. There were no obvious relationship between MICA and MICB and other clinical features such as sex, age, and distant metastasis (Additional file 1: Table S3).

### VPA enhances NK cell-induced lysis of pancreatic cancer cells

**Figure 1:**
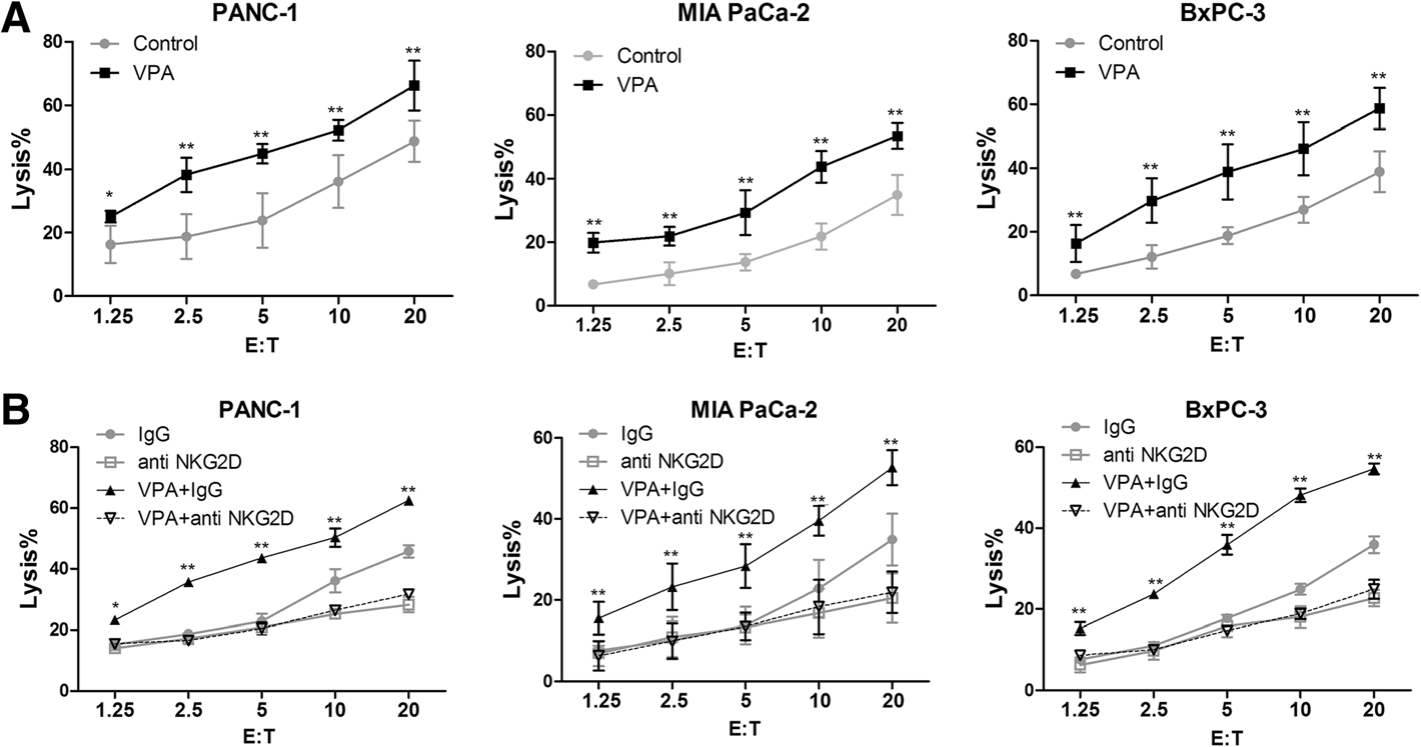
**VPA enhances the sensitivity of pancreatic cancer cells to NK cell-mediated lysis via a NKG2D-dependent mechanism. (A)** VPA sensitized pancreatic cancer cells to NK cell-mediated lysis; * *P* < 0.05; ** *P* < 0.01. **(B)** Blockade of NKG2D attenuated the ability of VPA to sensitize pancreatic cancer cells to NK cell-mediated lysis; * *P* < 0.05 for VPA + IgG vs. VPA + NKG2D; ** *P* < 0.01 for VPA + IgG vs. VPA + NKG2D. Data are mean ± SD of a single experiment performed in triplicate, all results were reproducible in three independent experiments. E:T, effector/ target ratio.

We first investigated the effect of VPA on NK cell-mediated kill of pancreatic cancer cells. PANC-1, MIA PaCa-2, and BxPC-3 cells were incubated with or without 1 mM VPA for 24 h. The LDH release assay demonstrated that NK-92 cells could lyse the pancreatic cancer cells; however, after incubated with 1 mM VPA for 24 hours, the lysis of PANC-1, MIA PaCa-2, and BxPC-3 cells mediated by NK-92 cells increased from 48.11% ± 8.29% to 66.22% ± 3.22%, 34.88% ± 4.09% to 53.11% ± 8.29% and 38.68% ± 4.09% to 58.81% ± 4.96% respectively at an effector/target (E/T) ratio of 20:1. The differences were statistically significant (Figure 1A). Pre-incubation of NK cells with an anti-NKG2D antibody for 30 minutes almost completely abolished the increased NK cell-mediated lysis of pancreatic cancer cells observed in VPA-treated co-cultures, indicating that the ability of VPA to promote the NK cell-mediated lysis of pancreatic cancer cells was dependent on a NKG2D/NKG2DL interaction between NK cells and pancreatic cancer cells (Figure 1B).

### VPA upregulates the expression of MICA and MICB in pancreatic cancer cells

**Figure 2:**
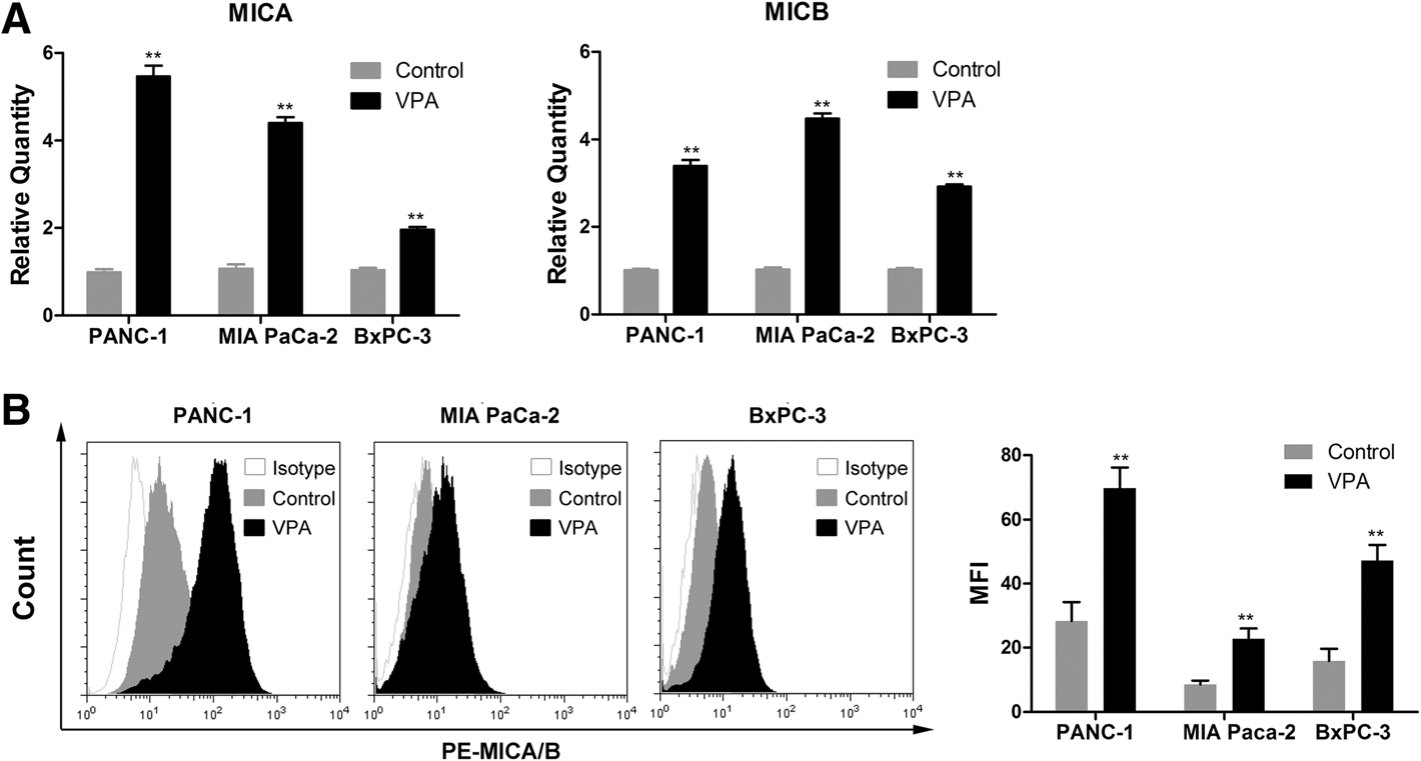
**VPA upregulates the expression of MICA and MICB in pancreatic cancer cells.** Pancreatic cancer cells were incubated with or without 1 mM VPA for 24 h. **(A)** Quantitative real-time RT-PCR analysis of *MICA* and *MICB* mRNA expression. **(B)** Flow cytometry analysis and quantification of MICA and MICB protein expression on the surface of pancreatic cancer cells. MFI, mean fluorescence intensity. Data are mean ± SD of a single experiment performed in triplicate, all results were reproducible in three independent experiments. ** *P* < 0.01.

The NKG2DLs MICA and MICB play an important role in the NK cell-mediated lysis of cancer cells [21]; therefore, we determined the effect of VPA on the expression of *MICA* and *MICB* mRNA in the human pancreatic cancer cell lines PANC-1, MIA PaCa-2, and BxPC-3. Real-time quantitative PCR analysis revealed that treatment with 1 mM VPA for 24 hours upregulated *MICA* and *MICB* mRNA expression significantly in PANC-1, MIA PaCa-2, and BxPC-3 cells (Figure 2A). We also examined the surface expression of MICA and MICB in pancreatic cancer cells treated with or without 1 mM VPA for 24 h. Flow cytometric analysis demonstrated that VPA significantly increased the expression of MICA and MICB on the cell-surface of PANC-1, MIA PaCa-2, and BxPC-3 cells (Figure 2B).

### VPA activates the PI3K/Akt pathway in pancreatic cancer cells

**Figure 3:**
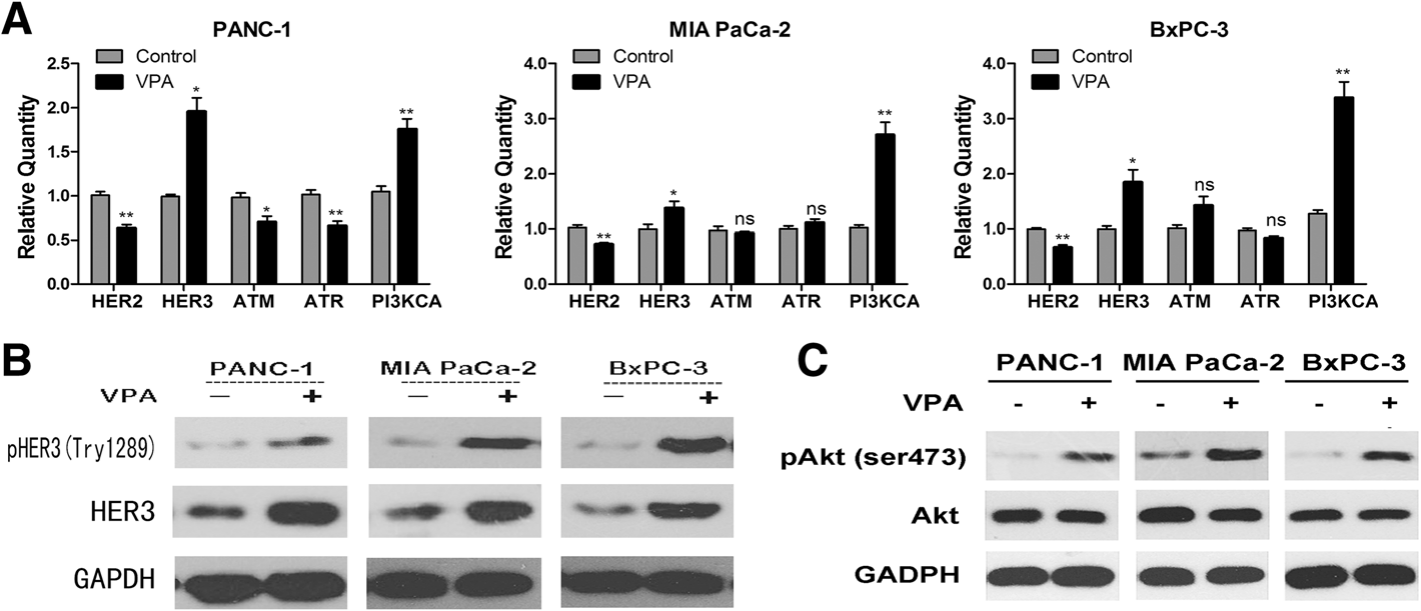
**VPA activates the PI3K/Akt signaling pathway in pancreatic cancer cells.** Pancreatic cancer cells were incubated with or without 1 mM VPA for 24 h. **(A)** Quantitative real-time RT-PCR analysis of *HER2, HER3, ATM*, *ATR*, and *PI3KCA* mRNA expression in PANC-1, MIA PaCa-2 and BxPC-3 cells. Data are mean ± SD of a single experiment performed in triplicate, all results were reproducible in three independent experiments. ns *P* > =0.05; * *P* < 0.05; ** *P* < 0.01. **(B, C)** Western blotting analysis of the expression and phosphorylation of HER3 and Akt (ser 473).

Expression of MICA and MICB are associated with a variety of signaling pathways, including the HER2/HER3, ATM/ATR, PI3K/Akt, and Erk pathways, in different cells [17,22-24]. To explore the mechanism by which VPA upregulates MICA and MICB in pancreatic cancer cells, we examined the expression and activation of components of the HER2/HER3, ATM/ATR, and PI3K/Akt pathways. Real-time quantitative PCR analysis revealed that VPA upregulated *HER3* and *PI3KCA,* and downregulated *HER2* in PANC-1, MIA Paca-2, and BxPC-3 cells. Additionally, VPA downregulated *ATM* and *ATR* in PANC-1 cells, but had no significant effect on *ATM* and *ATR* in MIA PaCa-2 and BxPC-3 cells (Figure 3A). Western blotting analysis revealed that incubation with 1 mM VPA for 24 h led to a significant increase in the expression and phosphorylation of HER3 protein (Figure 3B), as well as the phosporylated Akt in all three pancreatic cancer cell lines (Figure 3C), but not the phosphorylated Erk (Additional file 3: Figure S2).

### VPA-induced upregulation of MICA and MICB in pancreatic cancer cells is dependent on the PI3K/Akt pathway

**Figure 4:**
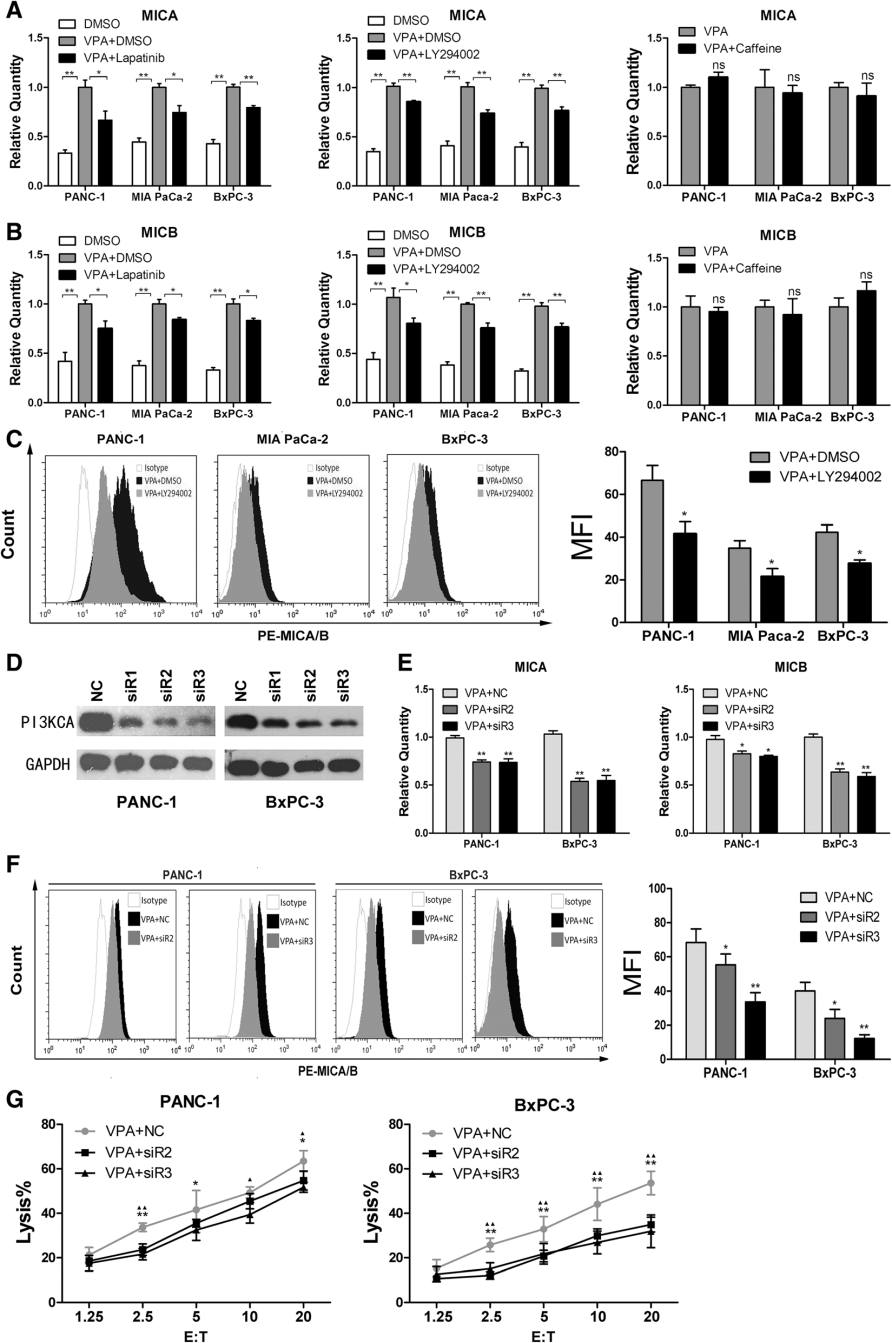
**PI3K/Akt signaling is required for VPA-induced upregulation of MICA and MICB in pancreatic cancer cells. (A, B)** Quantitative real-time RT-PCR analysis. The VPA-induced upregulation of *MICA* and *MICB* mRNA expression were inhibited by the HER2/HER3 inhibitor lapatinib and the PI3K inhibitor LY294002, but not by the ATM/ATR inhibitor caffeine. Data are mean ± SD of a single experiment performed in triplicate, all results were reproducible in three independent experiments. * *P* < 0.05; ** *P* < 0.01; ns *P* > 0.05. **(C)** Flow cytometry analysis. The VPA-induced upregulation of MICA and MICB protein expression on the cell surface were inhibited by the PI3K inhibitor LY294002. MFI, mean fluorescence intensity; * *P* < 0.05. **(D)** Western blotting analysis. Transfection of the PI3KCA siRNA inhibited PI3KCA protein expression at 48 h post-transfection. NC, negative control; siR1-3, PI3KCA_siR sequence 1-3; ** *P* < 0.01. **(E)** Quantitative real-time RT-PCR analysis. VPA-induced upregulation of *MICA* and *MICB* mRNA expression were attenuated in PI3KCA-knockdown cells; Data are mean ± SD of a single experiment performed in triplicate, all results were reproducible in three independent experiments. ** *P* < 0.01. **(F)** Flow cytometric analysis. VPA-induced upregulation of MICA and MICB protein expression on the cell surface were attenuated in PI3KCA-knockdown cells. MFI, mean fluorescence intensity; * *P* < 0.05; ** *P* < 0.01. **(G)** LDH release assay. The VPA-induced susceptibility of cancer cells to NK cell-mediated cell lysis was reduced in PI3KCA-knockdown cells. Data are mean ± SD of a single experiment performed in triplicate, all results were reproducible in three independent experiments. ** *P* < 0.01 and * *P* < 0.05 for NC vs. siR2; ▲▲ *P* < 0.01 and ▲ *P* < 0.05 for NC vs. siR3. siR2, PI3KCA siR sequence 2; siR3, PI3KCA siR sequence 3.

To determine whether the VPA-induced upregulation of MICA and MICB was related to activation of the HER2/HER3, PI3K/Akt, or ATM/ATR signaling pathways, PANC-1, BxPC-3, and MIA-Paca-2 cells were exposed to 1 mM VPA for 24 h in the presence or absence of 1 μM of the HER2/HER3 inhibitor lapatinib, 10 μM of the PI3K inhibitor LY294002, or 1 mM of the ATM/ATR inhibitor caffeine. Real-time quantitative RT-PCR and flow cytometric analysis demonstrated that the ability of VPA to upregulate the expression of MICA and MICB was significantly suppressed by lapatinib and LY294002, but not caffeine (Figure 4A-C). Next, we silenced PI3KCA using a siRNA in PANC-1 and BxPC-3 cells. Western blot analysis confirmed that the expression of PI3KCA was significantly reduced in PANC-1 and BxPC-3 cells 48 h after transfection of the siRNA (Figure 4D). Real-time quantitative RT-PCR and flow cytometric analysis demonstrated that the ability of VPA to upregulate the expression of MICA and MICB was significantly suppressed by transfection with PI3KCA siRNA (Figure 4E, F). Additionally, the ability of 1 mM VPA to increase the NK cell-mediated lysis of pancreatic cancer cells was significantly attenuated by knockdown of PI3KCA (Figure 4G). Although the role of PI3KCA siRNA on the expression of MICA and MICB protein was not totally compatible with its role on the NK cell-mediated lysis, the trend suggested that PI3K/Akt pathway played an important role in VPA-induced upregulation of MICA and MICB in pancreatic cancer cells.

### VPA improves the anti-tumor effects of NK-92 cells against pancreatic cancer xenografts in NOD/SCID mice

**Figure 5:**
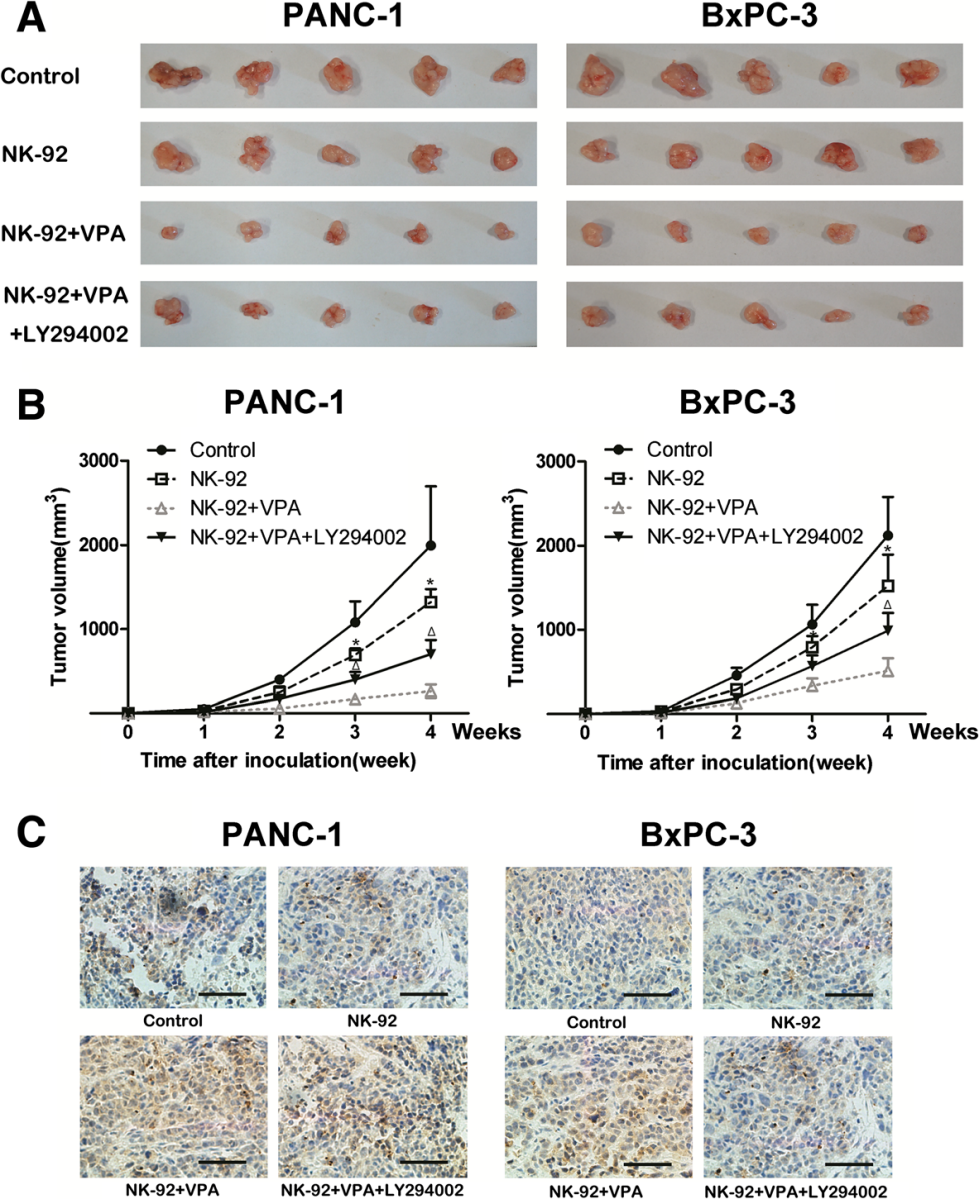
**VPA sensitizes pancreatic cancer cells to NK cell-mediated lysis *in vivo *by upregulating the expression of MICA and MICB. (A, B)** Excised xenograft tumors and growth curves for the xenografts; VPA enhanced the growth inhibitory effect of NK cells *in vivo*; this effect was attenuated by the PI3K inhibitor LY294002. Data are mean ± SD of five mice per group; * *P* < 0.05 for NK-92 vs. NK-92 + VPA; Δ *P* < 0.05 for NK-92 + VPA + LY294002 vs. NK-92 + VPA. **(C)** Immunohistochemical staining and quantification of MICA and MICB expression in the xenograft tumors; VPA significantly upregulated the expression of MICA and MICB in the pancreatic cancer xenograft tumors *in vivo*, this effect was attenuated by the PI3K inhibitor LY294002.

Results showed that treatment with VPA significantly enhanced the ability of NK-92 cells on inhibiting the growth of pancreatic cancer xenograft tumors; however, the anti-tumor effect of VPA was partly attenuated by treating the mice with the PI3K inhibitor LY294002 (Figure 5A, B). Furthermore, immunohistochemical analysis revealed that VPA significantly upregulated the expression of MICA and MICB in the tumor xenografts compared to the control group and NK-92 group, while administration of LY294002 significantly attenuated the ability of VPA on upregulation of MICA and MICB expression in the tumor xenografts (Figure 5C).

## Discussion

VPA, a histone deacetylase inhibitor which is used as an anti-epilepsy drug, was recently reported to exert anti-tumor effects by upregulating the expression of NKG2DLs, such as MICA/B and UL16-binding proteins (ULBPs), in a number of tumor types including hepatocarcinoma, myeloma, and myeloid leukemia [16,20,25-27]. These effects were linked to the activation of different signaling pathways in different tumor types, and were specific to malignant cells. In pancreatic cancer, the low expression of MICA was considered to be related to poor prognosis [28]. Our results revealed that the weak expression of MICA and MICB was correlated with worse tumor differentiation, later TNM stage, and more lymphatic invasion. The anti-tumor effects of VPA may have potential in the treatment of pancreatic cancer, for which there is currently no effective treatment. However, to our knowledge, there have been no reports on the effect and mechanism of action of VPA in pancreatic cancer.

In the present study, results suggested that 1 mM VPA did not inhibit the proliferation of pancreatic cancer cells (Additional file 4: Figure S3), but it enhanced NK cell-mediated lysis of pancreatic cancer cells, which relies on a NKG2D/NKG2DL-dependent interaction between NK cells and pancreatic cancer cells. MICA and MICB are important NKG2DLs which can effectively activate the NKG2D receptors and thereby induce NK cell-mediated cell kill [21]. Therefore, we analyzed the effect of VPA on the expression of MICA and MICB in pancreatic cancer cell lines. Our data revealed that the mRNA expression levels and cell surface expression of MICA and MICB were significantly upregulated by VPA.

In response to DNA damage, the expression of MICA and MICB can be induced by ATM and ATR, which are components of DNA damage signaling pathways [24,29,30]; these effects can be prevented by ATM/ATR inhibitors. In addition, MICA and MICB can also be induced by a variety of cell signaling pathways in different cell types; for example, HER2/HER3 signaling regulates the expression of MICA and MICB in human breast cancer cells [23]. Activation of Erk signaling increases the surface expression of MICA in myeloma cells, whereas inhibition of Erk signaling reduces the surface expression of MICA in ovarian tumor cells [17,22]. Additionally, transforming growth factor-beta (TGF-beta) selectively downregulates the expression of MICA, ULBP2, and ULBP4, but not MICB, ULBP1, or ULBP3, in malignant glioma cells [31].

To identify the signaling pathway involved in the VPA-induced upregulation of MICA and MICB in pancreatic cancer cells, the expression of a series of signaling molecules was analyzed using quantitative real-time RT-PCR. VPA downregulated *ATM* and *ATR* mRNA expression in PANC-1 cells, but had no significant effect on *ATM* and *ATR* in MIA PaCa-2 or BxPC-3 cells. Additionally, VPA upregulated the expression of *HER3* and *PI3KCA*, the gene which encodes the p110alpha catalytic subunit of PI3K [32], and downregulated *HER2* in PANC-1, MIA PaCa-2, and BxPC-3 cells. Western blotting analysis revealed that the expression and phosphorylation of HER3 were markedly increased by VPA, so does the phosphorylation of Akt, which suggested that VPA activates the HER2/3 - PI3K/Akt signaling pathway in pancreatic cancer cells. Additionally, lapatinib, an inhibitor of HER2/HER3 signaling [33], and the PI3K inhibitor LY294002 [23] inhibited the ability of VPA to upregulate MICA and MICB; whereas, caffeine, an ATM and ATR inhibitor [34] had no significant effect on the VPA-induced expression of MICA and MICB. These results demonstrated that HER2/HER3 signaling and its major downstream pathway, PI3K/Akt signaling, but not ATM/ATR signaling, are involved in the VPA-induced upregulation of MICA and MICB in pancreatic cancer cells.

We also validated the anti-tumor effect of VPA *in vivo* using a xenograft model of pancreatic cancer in NOD/SCID mice. In accordance with the *in vitro* experiments, VPA significantly enhanced the anti-tumor effect of NK cells against pancreatic cancer cells, as the tumors formed by VPA-treated pancreatic cancer cells were significantly smaller than those formed by untreated pancreatic cancer cells. In addition, the anti-tumor effect of VPA was significantly attenuated by administration of the PI3K inhibitor LY294002.

Activation of the PI3K/Akt pathway plays a vital role in the growth and survival of cancer cells. Consequently, several drugs targeting the PI3K/Akt signaling pathway have been developed to treat human cancer [35]. The PI3K inhibitor LY294002 has been proven exert an anti-cancer effect in a variety of tumor types both *in vitro* and *in vivo*[36-38]. It has been reported that LY294002 can inhibit the viability of MIA PaCa-2 pancreatic cancer cells to some extent [39], and increase the radiosensitivity of pancreatic cancer cells regardless of their *K-ras* mutation status [40]. However, the present study demonstrated that inactivation of PI3K using LY294002 or a siRNA attenuated the ability of VPA to upregulate the expression of MICA and MICB in pancreatic cancer cells. Our results suggest that inactivation of the PI3K signaling pathway may inhibit the immune effects of NK cells against pancreatic cancer cells, or at least inhibit the ability of VPA to enhance the anti-tumor effects of NK cells against pancreatic cancer cells. In addition, it must be pointed out that the plasma concentration of VPA in clinical use is usually 0.3-0.6 mM, which is a little lower than the concentration used in the present study. Thus some method for reducing their side effects should be developed before the clinical use of VPA for treatment of pancreatic cancer.

## Conclusions

Our results demonstrate that VPA enhances the susceptibility of pancreatic cancer cells to NK cell-mediated lysis by upregulating the expression of MICA and MICB on pancreatic cancer cells. Moreover, we provide evidence to confirm that the VPA-induced upregulation of MICA and MICB in pancreatic cancer cells is dependent on the PI3K/Akt signaling pathway. This data implies the potential of VPA in immunotherapy for patients with pancreatic cancer by upregulation of MICA and MICB. Considering the dependence of VPA effect on PI3K signaling activation, PI3K inhibitors should not be administered as anti-cancer drugs in patients with pancreatic cancer undergoing NK cell-mediated adoptive immunotherapy.

## Abbreviations

VPA: Valproic acid; HDAC: Histone deacetylase; NKG2D: Natural killer group 2D; MICA and MICB: Major histocompatibility complex class I-related chain A and B; PI3KCA: PI3K catalytic subunit alpha isoform; PDAC: Pancreatic ductal adenocarcinoma; BSA: Bovine serum albumin; FBS: Fetal bovine serum; DES: Donor equine serum; PE: Phycoerythrin; PBS: Phosphate buffer solution; HER2: Human epidermal growth factor receptor 2; HER3: Human epidermal growth factor receptor 3; ATM: Ataxia telangiectasia mutated kinase; ATR: ATM- and Rad3-related kinase; NC: Negative control; LDH: Lactate dehydrogenase.

## Competing interests

The authors declare that they have no competing interests.

## Authors’ contributions

PS was responsible for the manuscript preparation and most of the experimental work and results interpretation. SG and PC performed some experimental work. TY and SG participated in the study design and interpretation. CW and FZ were involved in the study design and supervision. All authors read and approved the final manuscript.

## Pre-publication history

The pre-publication history for this paper can be accessed here:

http://www.biomedcentral.com/1471-2407/14/370/prepub

## Supplementary Material

Additional file 1: Tables S1-S3 **Table S1.** The primers used in RT-PCR analysis; **Table S2.** The siRNA sequences used for PI3KCA knock down; **Table S3.** MICA and MICB expression and clinical characteristics of pancreatic cancer. Click here for file

Additional file 2: Figure S1 MICA and MICB expression in pancreatic cancer by immunohistochemical analysis. The antibody recognizes both MICA and MICB was used in the experiment. The positive staining for MICA and MICB was mainly distributed diffusely in the stroma of cancer cells in the duct-like structures. The MICA and MICB expression showed a decrease in poorly differentiated tumors. (A) Isotype control for immunohistochemical analysis. MICA and MICB expression in paracancerous tissues (B), well differentiated tumor (C), moderately differentiated tumor (D) and in poorly differentiated tumor (E). Click here for file

Additional file 3: Figure S2 Expression and phosphorylation of Erk in pancreatic cancer cells. 1 mM VPA treatment for 24 hours did not increase the phosphorylation of Erk in PANC-1, MIA PaCa-2 and BxPC-3 cells. Click here for file

Additional file 4: Figure S3 VPA has no significant effect on the proliferation of pancreatic cancer cells. PANC-1, MIA PaCa-2 and BxPC-3 cells were treated with 1 mM VPA for 24 hours, then cultured for 72 hours in normal medium. MTT assay show that there was no significant effect of VPA on the proliferation of PANC-1, MIA PaCa-2 and BxPC-3 cells. The result was reproducible in three independent experiments. ns *P* > =0.05. Click here for file
