# Supplementary material for: Valproic acid sensitizes pancreatic cancer cells to natural killer cell-mediated lysis by upregulating MICA and MICB via the PI3K/Akt signaling pathway
Source: BMC Cancer. 2014 May 25;14:370. doi: 10.1186/1471-2407-14-370 (PMC4076062; doi:10.1186/1471-2407-14-370)
Supplement: Additional file 1: Tables S1-S3 — Table S1. The primers used in RT-PCR analysis; Table S2. The siRNA sequences used for PI3KCA knock down; Table S3. MICA and MICB expression and clinical characteristics of pancreatic cancer. [file 1471-2407-14-370-S1.doc]

Table S1. The primers used in RT-PCR analysis

| primer | | sequence |
| --- | --- | --- |
| ACTIN | forward | 5’-GTCCACCGCAAATGCTTCTA-3’ |
|  | reverse | 5’-TGCTGTCACCTTCACCGTTC-3’ |
|  |  |  |
| MICA | forward | 5’-AAGACCAAGACACACTATCACGC-3’ |
|  | reverse | 5’-GGTGTCGTGGCTCAAAGATAC-3’ |
|  |  |  |
| MICB | forward | 5’-CTGATGGGAATGGAACCTACC-3’ |
|  | reverse | 5’-GTCTGTCCGTTGACTCTGAAGC-3’ |
|  |  |  |
| PI3KCA | forward | 5’-CTCCACGACCATCATCAG-3’ |
|  | reverse | 5’-TTCTTCACGGTTGCCTAC-3’ |
|  |  |  |
| HER2 | forward | 5’-GAGACCCGCTGAACAATACCA-3’ |
|  | reverse | 5’-CCTTCCACAAAATCGTGTCCTG-3’ |
|  |  |  |
| HER3 | forward | 5’-GTGCTCACGGGACACAATGC-3’ |
|  | reverse | 5’-GGAACCATCGGGAACTGACC-3’ |
|  |  |  |
| ATM | forward | 5’GCCAGACAGCCGTGACTTACT-3’ |
|  | reverse | 5’-CCAATACCTGTTTCTGAACCTCC-3’ |
|  |  |  |
| ATR | forward | 5’-TATCACCCAAAAGGCGTCGT-3’ |
|  | reverse | 5’-GCCACTGTATTCAAGGGAAATCT-3’ |

Table S2. The siRNA sequences for PI3KCA knock down

| siRNA | sequence |
| --- | --- |
| PI3KCA_siR1 | 5’-CCGTGAGGCTACATTAATA-3’ |
| PI3KCA_siR2 | 5’-GTAATTGAACCAGTAGGCA-3’ |
| PI3KCA_siR3 | 5’-GGTGAAAGACGATGGACAA-3’ |

**Table S3. MICA and MICB expression and clinical characteristics of pancreatic cancer**

|  | **Number of cases** | **MICA and MICB expression** | | **P** |
| --- | --- | --- | --- | --- |
|  |  | **Weak** | **Strong** |  |
| **Sex** |  | | | |
| *Male* | 45 | 26 | 19 | 0.802 |
| *Female* | 33 | 20 | 13 |  |
| **Age** |  | | | |
| *< 60* | 51 | 28 | 23 | 0.315 |
| *≥ 60* | 27 | 18 | 9 |  |
| **Differentiation** |  | | | |
| *Well* | 19 | 7 | 12 | 0.029 |
| *Moderately* | 38 | 24 | 14 |  |
| *Poorly* | 21 | 15 | 6 |  |
| **TNM stage** |  | | | |
| *I-II* | 42 | 20 | 22 | 0.028 |
| *III-IV* | 36 | 26 | 10 |  |
| **Lymphatic invasion** |  | | | |
| *Positive* | 52 | 35 | 17 | 0.034 |
| *Negative* | 26 | 11 | 15 |  |
| **Distant metastasis** |  | | | |
| *Positive* | 19 | 10 | 9 | 0.518 |
| *Negative* | 59 | 36 | 23 |  |
