# Supplementary figures and images for: Valproic acid sensitizes pancreatic cancer cells to natural killer cell-mediated lysis by upregulating MICA and MICB via the PI3K/Akt signaling pathway
Source: BMC Cancer. 2014 May 25;14:370. doi: 10.1186/1471-2407-14-370 (PMC4076062; doi:10.1186/1471-2407-14-370)

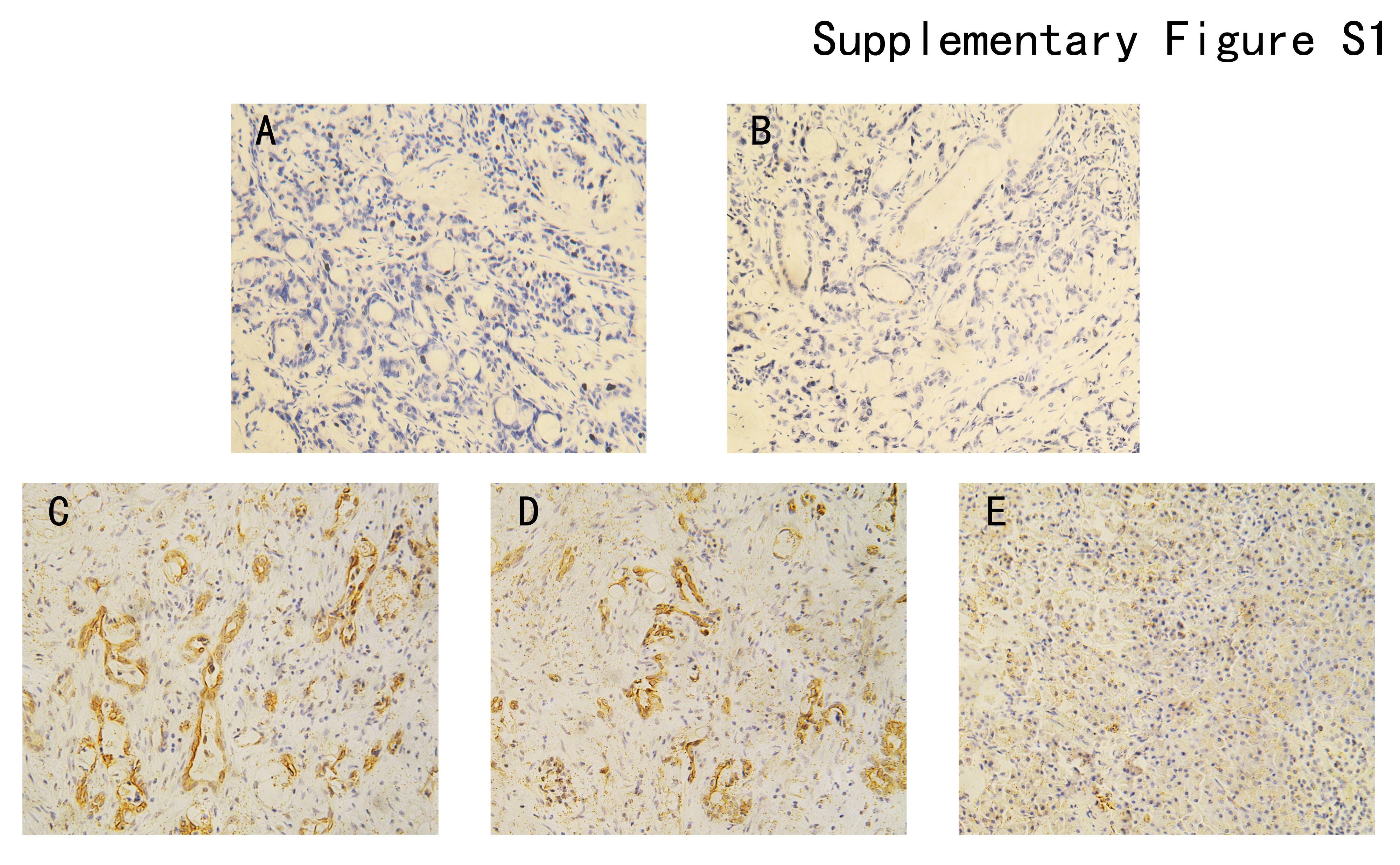

Supplement: Additional file 2: Figure S1 — MICA and MICB expression in pancreatic cancer by immunohistochemical analysis. The antibody recognizes both MICA and MICB was used in the experiment. The positive staining for MICA and MICB was mainly distributed diffusely in the stroma of cancer cells in the duct-like structures. The MICA and MICB expression showed a decrease in poorly differentiated tumors. (A) Isotype control for immunohistochemical analysis. MICA and MICB expression in paracancerous tissues (B), well differentiated tumor (C), moderately differentiated tumor (D) and in poorly differentiated tumor (E). [file 1471-2407-14-370-S2.tiff]

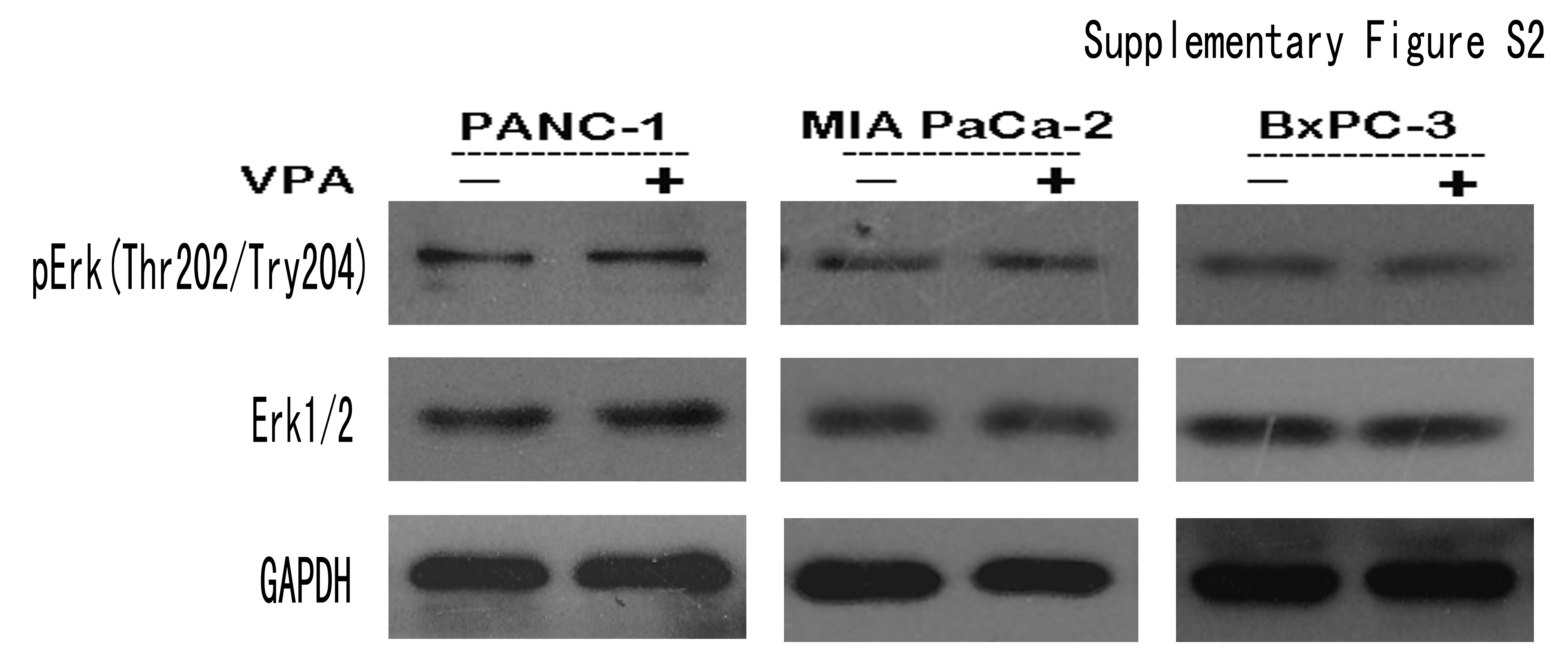

Supplement: Additional file 3: Figure S2 — Expression and phosphorylation of Erk in pancreatic cancer cells. 1 mM VPA treatment for 24 hours did not increase the phosphorylation of Erk in PANC-1, MIA PaCa-2 and BxPC-3 cells. [file 1471-2407-14-370-S3.tiff]

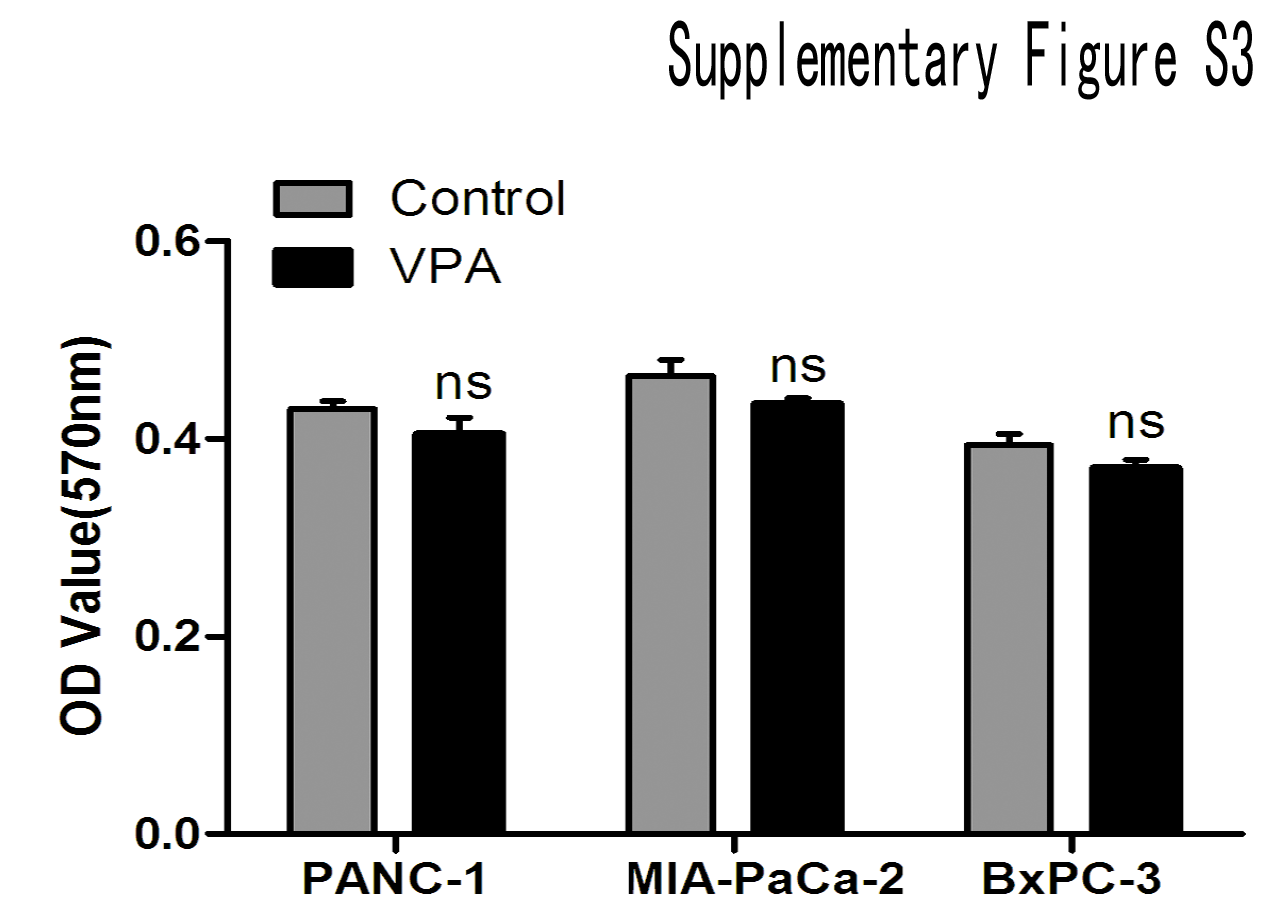

Supplement: Additional file 4: Figure S3 — VPA has no significant effect on the proliferation of pancreatic cancer cells. PANC-1, MIA PaCa-2 and BxPC-3 cells were treated with 1 mM VPA for 24 hours, then cultured for 72 hours in normal medium. MTT assay show that there was no significant effect of VPA on the proliferation of PANC-1, MIA PaCa-2 and BxPC-3 cells. The result was reproducible in three independent experiments. ns P > =0.05. [file 1471-2407-14-370-S4.tiff]
